# A model-specific simplification of the Mouse Grimace Scale based on the pain response of intraperitoneal CCl_4_ injections

**DOI:** 10.1038/s41598-022-14852-0

**Published:** 2022-06-28

**Authors:** Lisa Ernst, Stefan Bruch, Marcin Kopaczka, Dorit Merhof, André Bleich, René H. Tolba, Steven R. Talbot

**Affiliations:** 1 Faculty of Medicine, Institute for Laboratory Animal Science and Experimental Surgery, RWTH Aachen University, Aachen, Germany; 2 Institute of Imaging and Computer Vision, RWTH Aachen University, Aachen, Germany; 3 Institute for Laboratory Animal Science, Hannover Medical School, Hanover, Germany

**Keywords:** Zoology, Animal behaviour, Animal physiology, Experimental models of disease

## Abstract

Despite its long establishment and applicability in mice pain detection, the Mouse Grimace Scale still seems to be underused in acute pain detection during chronic experiments. However, broadening its applicability can identify possible refinement approaches such as cumulative severity and habituation to painful stimuli. Therefore, this study focuses on two main aspects: First, five composite MGS criteria were evaluated with two independent methods (the MoBPs algorithm and a penalized least squares regression) and ranked for their relative importance. The most important variable was used in a second analysis to specifically evaluate the context of pain after an *i.p.* injection (intervention) in two treatment groups (CCl_4_ and oil (control)) at fixed times throughout four weeks in 24 male C57BL/6 N mice. One hour before and after each intervention, video recordings were taken, and the MGS assessment was performed. In this study, the results indicate orbital tightening as the most important criterion. In this experimental setup, a highly significant difference after treatment between week 0 and 1 was found in the CCl_4_ group, resulting in a medium-sized effect (W = 62.5, *p* value < 0.0001, r_CCl4_ = 0.64). The oil group showed no significant difference (week 0 vs 1, W = 291.5, *p* value = 0.7875, r_control_ = 0.04). Therefore, the study showed that the pain caused by *i.p.* injections was only dependent on the applied substance, and no significant cumulation or habituation occurred due to the intervention. Further, the results indicated that the MGS system can be simplified.

## Introduction

The EU Directive 2010/63 protects animal life and welfare when animals are used in experiments, e.g., biomedical research^1^. When using animals, the aim should always be the greatest possible well-being and the reduction of animal suffering through pain, distress, or harm. When assessing severity, pain recognition is one major factor to be considered^2^. The perception of pain varies between individuals, but it can also be shown in various ways regarding the different animal species. In this context, facial expressions are an example of showing pain in certain animals, e.g., rodents^3^. The pain face, or, so-called grimace scale, which was initially developed in humans for the recognition of pain in children or other patients who depend on non-verbal communication^4^, is scaling the pain sensation based on the expression of different facial features. Meanwhile, the Mouse Grimace Scale (MGS)^5^ was developed and transferred to different animal species as well^6–11^. Numerous studies demonstrated and verified the applicability and utilization of the grimace scale for pain recognition^12,13^. The following animal-specific facial criteria, also known as Facial Action Units (FAU) are taken into account: Orbital tightening (OT), ear posture (EP) , cheek bulge (CB), nasal wrinkling (NB) and whisker change^5^. These 5 criteria are scored by observers and classified into degrees of deviations as a function of severity classes. The summation allows a classification of the animal at the specific time to a degree of pain. All criteria are equally weighted in this approach.

The application of the grimace scales in laboratory animal science is intended to provide the possibility of classifying specific interventions and treatments and ensure better medical care for the animals within the experiment through the direct assessment of the pain condition. This means that the MGS can also be used directly as a target for possible refinement measures in the context of the 3R-principles^14^.

Despite the method’s ease of accessibility^11^, the Mouse grimace scale has not yet been widely used on a routine basis for performing basic, day-to-day severity assessments during experiments. Most studies that used the grimace scale were either focusing on evaluating the MGS system^15,16^ itself using different techniques or settings or had pain detection and assessment as a direct scientific focus^17,18^.

The studies investigating the grimace scale's applicability showed that time and personnel requirements still impeded its extensive use and, above all, a direct on-site approach due to its retrospective evaluation character^16,19^. In addition to the general ease of application, the MGS method^5^ showed good inter-rater variability^15,20^. However, inter-individual variations in the particular assessment criteria or action units and the influence of subjective perceptions on the assessment can still result in further difficulties in the usability of this method^21^.

In our opinion, these standardization problems can lead to the conclusion that the application is too intricate or too extensive in its basic structure to achieve precise results.

Our study aimed to characterize the five MGS examination criteria and their contribution to the overall scoring. Further, we analyzed how changes in the examination criteria or singularization influenced the final scoring. With these insights, we hypothesize that changes or singularization in the examination criteria facilitate changes in the animals’ pain face scoring. The MGS examinations were performed as an evaluation of the pain assessment following repeated *i.p.* injections (intervention) with CCl_4_ or oil (treatment) at predefined regular intervals. Therefore, the resulting pain stimulus was classified with the MGS.

## Materials and methods

### Ethical statement

This animal study was approved by the Governmental Animal Care and Use Committee of the federal state of North Rhine-Westphalia (LANUV, North Rhine-Westphalia, Germany) (Protocol No. AZ: 84-02.04.2014.A417). The study protocol complied with the EU Directive 2010/63 and the Guide for the Care and Use of Laboratory Animals^22^. This study was performed in accordance with the application of the 3Rs criteria as a branch project from a recently published animal study on evaluation severity assessment in fibrosis induction^23^. The animals were examined retrospectively, no additional experiments were carried out. The study was performed and reported in accordance with the ARRIVE guidelines^24^.

### Animals and study design

Twenty-four male C57Bl/6 N animals (Janvier, France) of approximately 8 weeks of age were used. During the experiment, the animals were kept in a controlled spf barrier according to the FELASA recommendations^25^. Humane endpoints were set at each stage of the study to avoid severe pain, harm, or distress of the animals. These animals were weighed and then divided randomly (randomizer.org) into two treatment groups: A CCl_4_ group and a control group (oil) for further investigation in a liver fibrosis model^23^. For this purpose, the animals were injected *i.p.* with 50 µl of the treatment solution three times a week over 4 weeks (Monday, Wednesday, and Friday). The MGS examination was carried out on these treatment days according to a set-up that we have recently published^15^. Briefly, the animals were filmed in an MGS observation box for 10 min. The observation box was placed in their home cage for handling animals. Then individual animals were gently lifted and placed into the observation box. The filming was carried out 1 h before the injection and exactly 1 h after the injection of the respective animal. To investigate the effect of the intervention (= injection) between the different treatment groups, the animals were observed at the same daytime on the intervention days. At each time point, eight images were randomly selected in each video by the algorithm^15^. Subsequently, these pictures were issued blindly and manually evaluated by the investigator (> 4 years of experience in laboratory animal science) within this study. According to the ARRIVE guidelines, additional information concerning housing and husbandry conditions can be found in the supplementary material.

### Data science and analysis

Statistical analysis and data evaluation were performed using the R software (v4.0.3^26^) and the recently published algorithm for identification of the best performing variable by data-mining and cooperative game theory for evaluating study criteria (MoBPS = mining on best parameter search)^27^). Data were grouped and summarized using the dplyr^28^ package. Distributions were tested with quantile–quantile plots and Shapiro Wilk’s test. In the case of non-Gaussian or mixed distributions, 10,000-fold bootstrapping was applied to obtain the median estimates and 95% confidence intervals (CI) (boot^29^). Raw data are available at https://github.com/mytalbot/MGS_data.

To explore the variables’ impact on the average picture score, two independent strategies were followed. In the first approach, the five independent criteria (orbital tightening (OT), nose bulge (NB), cheek bulge (CB), ear position (EP), and whisker change (WC)) were analyzed with the MoBPS algorithm.

MoBPS examines the ability of parameter combinations to quantify intervention effects between pre-and post-intervention conditions of treatment groups. The assumption is that multivariate measures can have greater explanatory power than single variables. Measures of univariate comparisons of treatment groups are statistical effect sizes. MoBPS modifies effect sizes to make groups of different sizes and distribution comparable and creates a multi-parameter measure M. This M is determined for each possible combination and normalized to the maximum occurring value M_max_. Also, the effect of each parameter on the overall measure was determined using a Shapley value.

In a second approach, a generalized linear model with a penalized maximum likelihood (glmnet) was applied^30^, in which the average picture score was modeled as a function of the highly correlated grimace scale criteria and their interactions with time (“week”) and intervention (“pre/post”) using tenfold cross-validation and a least absolute shrinkage and selection operator (LASSO) (α = 1) to ensure the robustness of the coefficients. The most parsimonious model within one standard error of the best-performing model was used to select the coefficients. This was calculated independently in each treatment group (control (“Oil”) and “CCl_4_”). Week 0 was excluded due to rank deficiency of the intervention variable (intervention started in week 1). The input variables were scaled so that the resulting coefficients could be ranked and compared.

The “most meaningful” dependent variable from the MGS ensemble was tested for both, the *between-treatments* and *within-treatment* contrasts. Further, two different time resolutions (day and weeks) were tested. The change of default levels for these contrasts made it necessary to restructure the model for the analyses, e.g., to assess the specific coefficients in each treatment separately (Supplemental Material S1–2 for more information). The independent variables (treatment, day/week, and intervention) were set as fixed effects (FE) and interactions. In total, three models were used in the analysis: (I) a generalized *between-treatments* model at the highest available time resolution (day) and with day nested in weeks as random effects (RE), (II) a *within-treatment* model of CCl_4_, excluding data from week 0 to avoid rank deficiency for the missing intervention data, (III) same as (II) but with the control group. The models were calculated as linear mixed-effects regressions (lmer (lme4^31^, lmerTest^32^)) using the animal ID as random effects (RE) in a random intercepts model with the restricted maximum likelihood estimator. The Kenward-Roger's approximation of the degrees of freedom was used to calculate the confidence intervals and *p* values of the mixed models.

To assess the impact of the intervention variable on animal welfare and baseline differences, a Mann–Whitney U test was used to test whether there was a difference between animals in week 0 without an intervention (“bsl = baseline”) and after an intervention (“post”) on week 1. This was performed in both treatment groups (control and CCl_4_) under the alternative hypothesis that the true location shift was not equal to 0.

Further, group differences in time-independent cumulative severity counts were determined with a χ^2^-square test. Finally, post-hoc tests were calculated with the rcompanion^33^ package to adjust for multiple comparisons.

Results with *p* ≤ 0.05 were considered significant in all inferential tests. In all examinations ,the grimace scale was discretized into classes of no, moderate or severe deviations from the physiological situation in analogy to the current publications^5,11,34^. This was followed by a retrospective arbitrary assignment of severity at the following thresholds [Score Level = MGS < 3: mild; MGS > = 3 and < = 6: moderate; MGS > 6: severe] in alignment with the severity levels of this model shown in 2020^23^.

## Results

### Variable importance and selection

To analyze the severity of the intervention based on the MGS image scores, a total of 4944 images (average of 8 pictures/animal/video) were randomly selected for evaluation using a picture selection tool similar to our previous studies^15^. Of these images, 749 could not be included because of poor quality or non-recognizability (are marked as − 1 = rejected in the raw data) of the evaluation criteria (e.g., whisker change). Data were integrated for mean values in terms of repeated measurements from different video sources. Further, in addition to the five MGS criteria, the time resolution of the measurements was noted in two variables, “week” (0, 1, 2, 3, 4) and “day” (day 1, 2, and 3) as well as the variables treatment (Oil, CCl_4_), intervention (baseline, pre, and post), and animal ID. The final data set had the dimensions of 498 rows with n = 24 unique animal identifiers.

**Figure 1:**
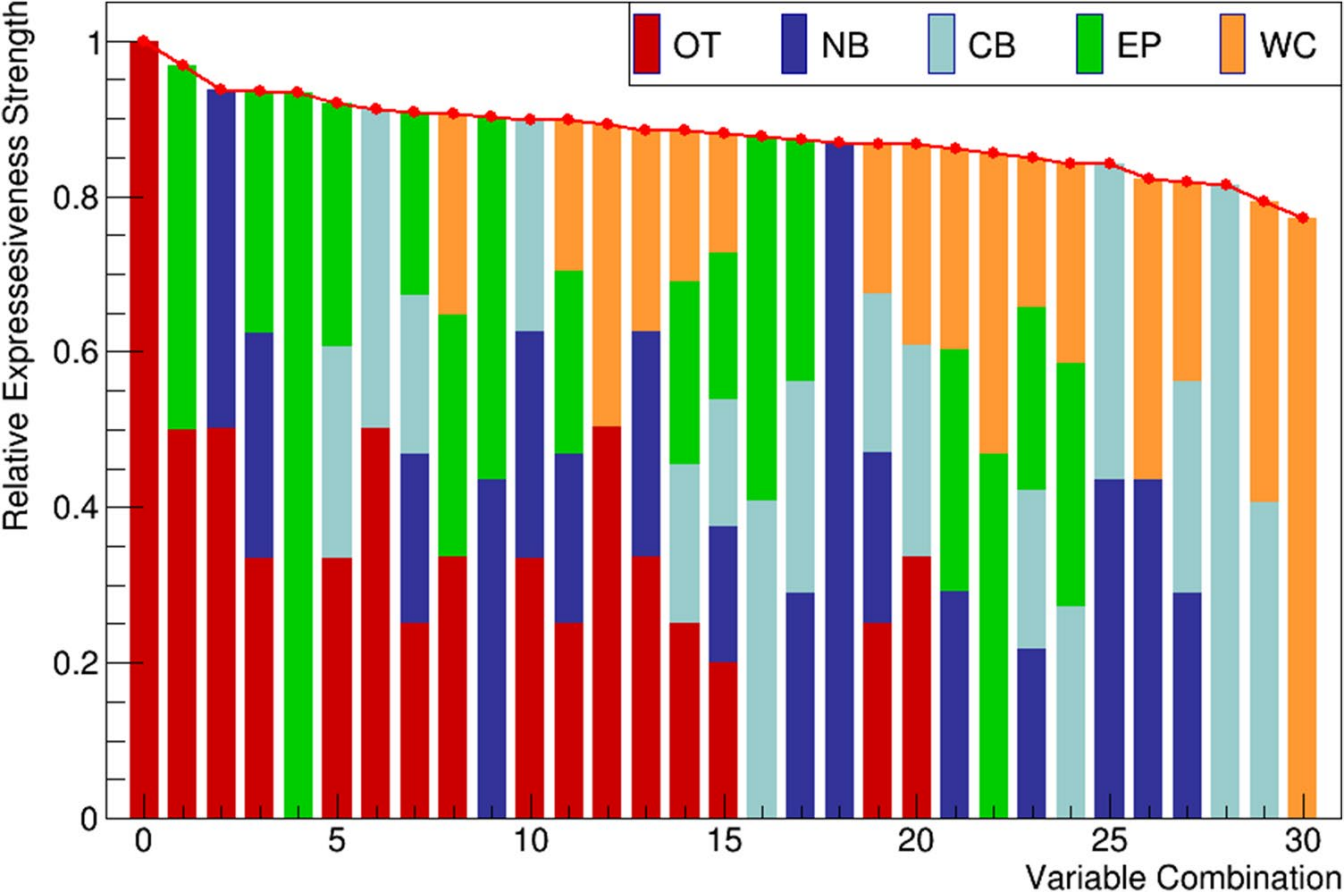
Strength of expressiveness for all combinations of the scoring criteria: (orbital tightening (OT), nose bulge (NB), cheek bulge (CB), ear position (EP), and whisker change (WC)) considering the intervention (injection). The red points indicate the combination importance strength relative to the maximum value, the bars indicate the criterias' weight within the combination.

Initially, the priority of the different MGS evaluation criteria was determined with the MoPBs algorithm. As a result, the expressiveness of specific parameters was ranked and quantified relative to the most meaningful value (defined as 100%). Figure 1 shows the result of these analyses and identifies orbital tightening as the first-ranked parameter and whisker change as the last-ranked parameter. Further, the algorithm explored criteria combinations like OT and NB as second best, etc.

In addition to the expressiveness, time- and intervention-independent correlations of the grimace scale criteria in each treatment group were analyzed. Highly correlated parameters in (Table 1) are showing the same impact on the MGS outcome. Orthogonal parameters can be substituted with each other. The overall correlations in the CCl_4_ group were higher than in the Oil group. In both treatment groups, the NB ~ CB combination shows the highest correlation of all criteria (Oil, *r*_NB~CB_ = 0.817; CCl_4_, *r*_NB~CB_ = 0.901).

**Table 1:** Time- and intervention-independent correlations of the grimace scale criteria.

	Orbital tightening	Nose bulge	Cheek bulge	Ear position	Whisker change
**Oil**					
Orbital tightening	1				
Nose bulge	0.718	1			
Cheek bulge	0.728	0.817	1		
Ear position	0.572	0.707	0.777	1	
Whisker change	0.615	0.78	0.812	0.81	1
**CCl**_**4**_					
Orbital tightening	1				
Nose bulge	0.876	1			
Cheek bulge	0.898	0.901	1		
Ear position	0.836	0.835	0.848	1	
Whisker change	0.801	0.843	0.855	0.861	1

**Figure 2:**
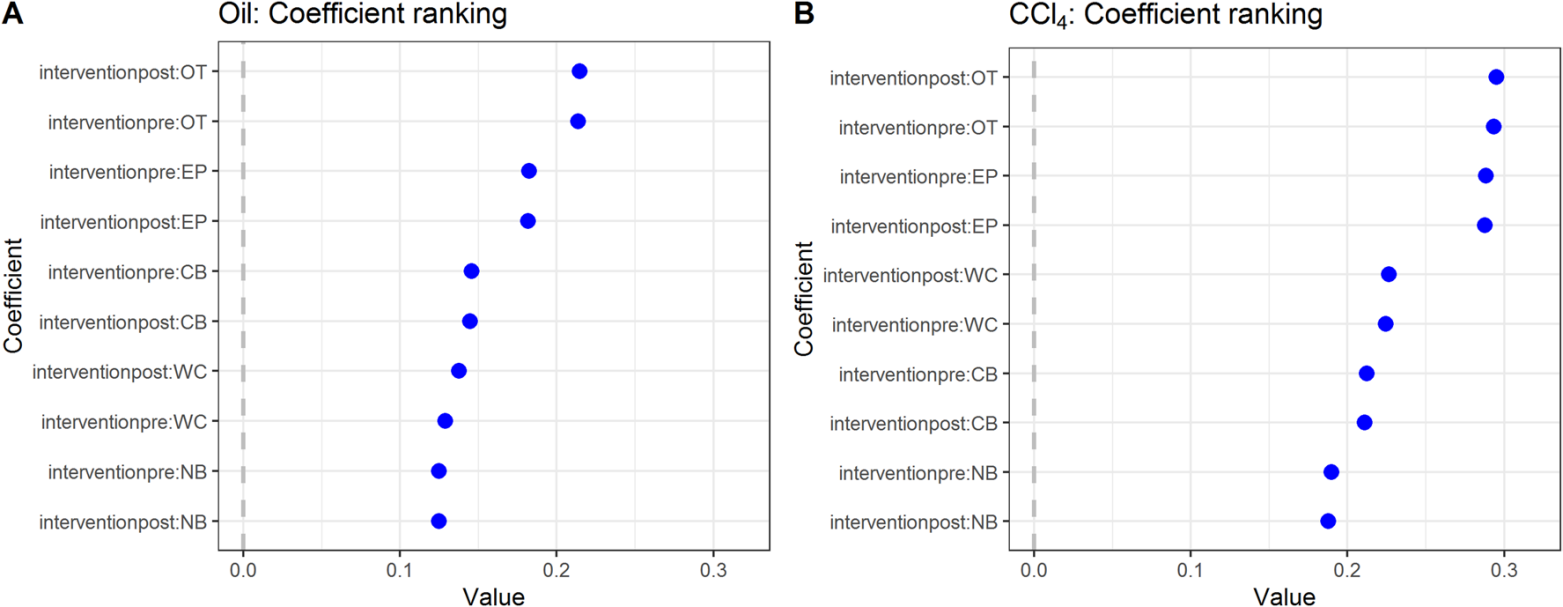
Time-independent regression coefficients of the penalized least square regression, ordered by magnitude. The coefficients describe the relationship between the predictor variables and the response (MGS). Larger coefficients have more weight in the regression model and are deemed more important. (**A**) In the control group, the orbital tightening parameter shows the largest and nose bulge the lowest coefficients. (**B**) In the CCl_4_ group, all coefficients have higher values, and, orbital tightening is also the highest-ranking coefficient found by the model. Nose bulge performed worst.

In general, however, the results show that all parameters are highly correlated and, therefore, show strong collinearity in regular regression analysis. To compensate for this, we used a penalized maximum likelihood regression capable of both variable selection and regularization of the model. We used tenfold cross-validation to minimize the mean squared error on the λ estimator *(λ*_1SE,Oil_ = 0.001, *λ*_1SE, CCL4_ = 0.306). Figure 2 shows the result of the coefficient ranking from the LASSO regression. A time-independent analysis showed that the orbital tightening parameter in both treatment groups and interventions had the largest values *β*_CCL4,OT,post_ = 0.295, *β*_CCL4,OT,pre_ = 0.293, compared to *β*_Oil,OT,post_ = 0.215, *β*_Oil,OT,pre_ = 0.214. Interestingly, the second strongest parameter in both treatment groups was found to be the EP parameter (*β*_CCL4,EP,pre_ = 0.289, *β*_CCL4,EP,post_ = 0.288, compared to *β*_Oil,OT,pre_ = 0.182 and *β*_Oil,OT,post_ = 0.182). Although not a combination of parameters, this is similar to the findings of the MoBPs algorithm, where the second-best full parameter is also ear position (Fig. 1, full green bar). However, in terms of the weakest contributing variable, the two methods showed different results. The MoBPs algorithm finds whisker change as the worst-performing variable, while the LASSO regression finds nose bulge, again in both treatment methods. In the regression model, whisker change performed better than cheek bulge in the CCl_4_ group. In the control group, this was reversed.

Due to the overall agreement of the high applicability of the orbital tightening in our results and the simultaneous easy recognizability for future automated examination procedures, we have identified the orbital tightening as a potential "target parameter" for subsequent examinations.

### The regression model of the OT analysis

In the second part of the analysis, multiple linear mixed regression models with orbital tightening as the dependent variable were built to analyze different treatments and interventions over time affecting the orbital tightening variable (Table 2). The main target factor is investigating the effects of the parameter OT on the treatment, the intervention, and the time.

**Table 2:** Overview of the regression models with Fixed Effects (FE) and Random Effects (RE) parameters.

Model	FE	RE	Type
I	Treatment: day: intervention	Animal ID & week/day	Between-treatments
II	Week: intervention	Animal ID	Within-treatment CCl_4_
III	Week: intervention	Animal ID	Within-treatment Oil

### Model I: orbital tightening between-treatments analysis

**Figure 3:**
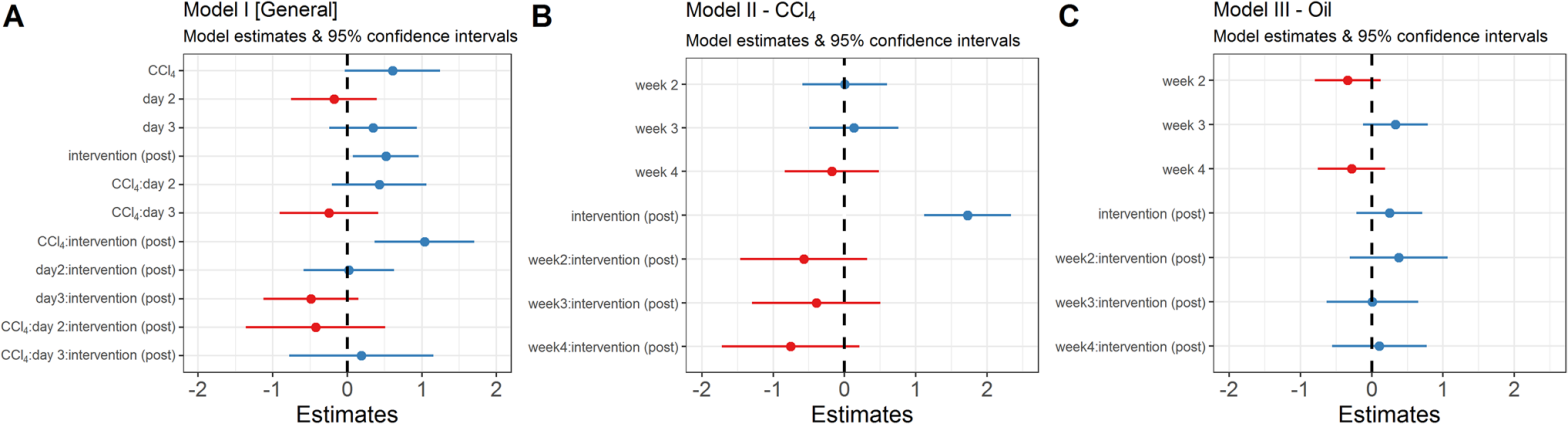
Coefficient estimates with 95% confidence intervals from linear the mixed-effects regressions of the orbital tightening variable (red/blue color: negative/positive coefficients). (**A**) General between-treatments model (default levels were Oil, day 1, and intervention (pre)) with significant coefficients for intervention (post) and CCl_4_:intervention (post). (**B**) Within-CCl_4_ data over weeks (default level week = 1). No significant coefficients for week:intervention were found but there was evidence for a negative slope indicating habituation. Intervention, in general, was different from the default level in the CCl_4_ group. (**C**) No significant coefficients were found in the control group (Oil).

In model I (Supplemental Material S2–3), the highest available time resolution “day” was included in an interaction with the “intervention” variable and the “treatment” groups (Oil and CCl_4_). The *between-treatments* model (I) with animal ID as RE was extended by a random intercept term in which “day” was nested within the “week” variable (*β*_*Intercept*_ = 2.59, CI_95%_[2.04; 3.14], *p* < 0.001). From the total variance, the animal ID was able to explain 21.56% (τ_ID_ = 0.32), the interaction day:week 5.33% (τ_day:week_ = 0.08) and week 0.77% (τ_week_ = 0.01) of the variance in the data. The remaining unexplained variance remained high with 72.33% (σ^2^ = 1.09). With the *between-treatments* model (I), no significant difference between treatment groups was found. However, there was evidence for a potential difference (*β*_CCl4_ = 0.601, CI_95%_[− 0.05; 1.26], *p* = 0.069). Compared to the given default levels in the oil group, CCl_4_ showed higher values in orbital tightening (*β*_*Intercept*_ = 2.59 + *β*_CCl4_ = 0.601 = 3.191). Despite this large estimate, the effect was not significant at the α = 0.05 level and the given variance. The model found a significant general difference for the “intervention” predictor between treatments (*β*_CCl4_ = 0.52, CI_95%_[0.07; 0.96], *p* = 0.022). Post-intervention was significantly higher than pre-intervention in terms of the time- and treatment-independent intervention effect. This difference was most prominent in the CCl_4_:intervention interaction, when compared to the default levels of the treatment-model (*β*_CCl4:intervention_ = 1.03, CI_95%_[0.36; 1.7], *p* = 0.003). While the *between-treatments* predictor was not significant, the interaction with intervention shows that CCl_4-_post-intervention was higher than Oil-pre-intervention. In model I, “day” or its interactions with “treatment” or “intervention” did not show significant differences (Fig. 3A).

### Model II: orbital tightening within-CCl_4_ analysis

The analysis in model II focused on CCl_4_ data (Supplemental Material S2, S4). Here, the *within-treatment* development of severity over time was modeled. Therefore, baseline data (at week 0) with missing interventions were excluded. As a result, this model's default level of “week” was 1. Baseline level comparisons are shown in model I. Orbital tightening was modeled as a function of the interaction terms “week” and “intervention” (*β*_*Intercept*_ = 3.30, CI_95%_[2.77; 3.83], *p* < 0.001) with animal ID as random effects. The animal ID was able to explain 24.51% (τ_week_ = 0.341) of the model variance. The residual variance remained high at 75.49% (σ^2^ = 1.392). Compared to the default levels, only the time-independent “intervention” predictor was significant (*β*_intervention_ = 1.73, CI_95%_[1.12; 2.34], *p* < 0.001). Thus, an intervention increased the orbital tightening value from 3.3 to 5.03 units. No other *within-treatment* coefficient or interaction with “week” was significant (Fig. 3B). Nevertheless, the week:intervention estimates in the model showed a continuous decrease over time, indicating a return of the orbital tightening values towards the default levels (week 1, pre-intervention) (*β*_week2:intervention_ = − 0.57, CI_95%_[− 1.47; 0.33], *p* = 0.21; *β*_week3:intervention_ = − 0.40, CI_95%_[− 1.31; 0.51], *p* = 0.391; *β*_week4:intervention_ = − 0.75, CI_95%_[− 1.73; 0.22], *p* = 0.22).

### Model III: orbital tightening within-oil analysis

In the third model (III), baseline data were excluded in the same way as in model II (Supplemental Material S2, S5). The orbital tightening was modeled as a function of the interaction terms “week” and “intervention” (*β*_*Intercept*_ = 2.70, CI_95%_[2.24; 3.15], *p* < 0.001) with animal ID as random effects. The animal ID was able to explain 29.80% (τ_week_ = 0.283) of the model variance. The residual variance remained high at 70.20% (σ^2^ = 1.392). No significant coefficients were found (Fig. 3C). The week:intervention coefficients remained inconclusive of a trend and were small (*β*_week2:intervention_ = 0.38, CI_95%_[− 0.31; 1.07], *p* = 0.282; *β*_week3:intervention_ = 0.01, CI_95%_[− 0.64; 0.66], *p* = 0.978; *β*_week4:intervention_ = 0.11, CI_95%_[− 0.56; 0.77], *p* = 0.754), indicating no intervention effect in general or over time.

### Severity classification and pain assessment

**Figure 4:**
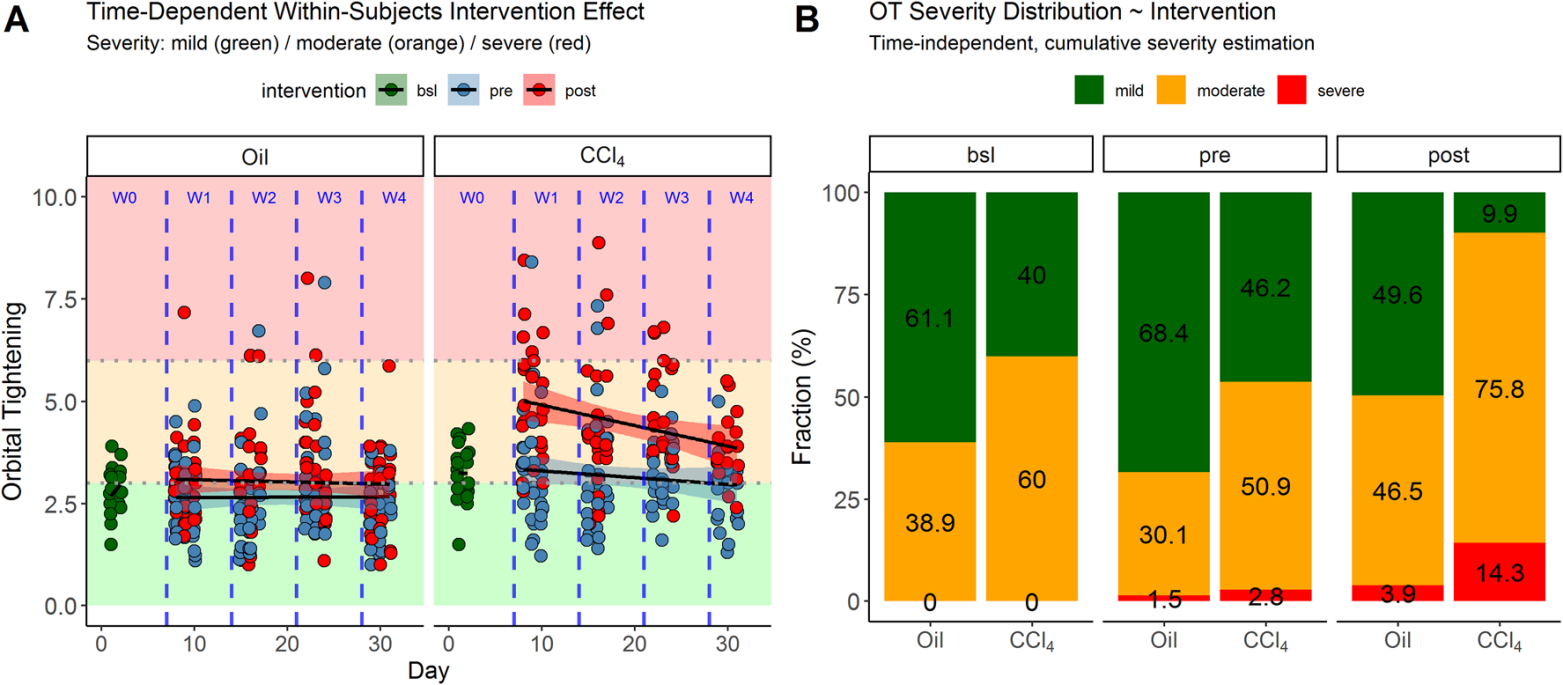
(**A**) Distribution of orbital tightening over time contrasted by the within-subjects intervention regimes pre (steel blue)/post (red) in the two treatment groups. The untreated baseline values are shown in week 0 (dark green). Note that in week 1 the animals show higher values after the intervention (red) than in week 0. These differences were not prominent in the control group. Further, the grimace scale thresholds are shown as colored regions on the y-scale (green = mild, orange = moderate, red = severe). In the CCl_4_ group, more animals were found in the moderate and the severe classes than in the control group. In the CCl_4_ group, the animals show an elevated baseline (60% of the CCl_4_ animals in week 0, compared to 38.9% in the Oil group). Further, the fraction of severity was increased in both treatment groups after the intervention. (**B**) Time-independent cumulative severity estimation. The number of animals in each severity class was counted and expressed as a percentage (fraction). The severity classes are colorized as in (**A**).

Figure 4A shows the time-dependent group contrasts in the treatment groups, colorized by *within-subjects* differences of interventions. Notably, the variance was high in all contrasts. The regression models I-III have shown large amounts of variance in the groups that cannot be explained with any of the experimental variables. The resulting intra-class correlation coefficients were, therefore, small (ICC_I_ = 0.28, ICC_II_ = 0.20, ICC_III_ = 0.23).

In the control group, the median development of the post-interventional severity was not as high as it was in the CCl_4_ group (see “intervention (post)” in models II and III, Fig. 3B and C). Both treatment groups started at different baseline values (bootstrapped estimates: Oil_week0_ = 2.76, CI_95%[_2.37; 3.16], and CCl_4,week0_ = 3.25, CI_95%_[2.74; 3.76]). This difference was significant (W = 105, *p* = 0.029). Further, the distribution of data into the three discretized severity classes was also different in the group comparisons. CCl_4_ showed more directionality towards higher severity in the post-intervention group (red points in the red area) than the control group. Figure 4B explores the cumulative and time-independent development of severity in the data. For this, data in the discrete classes were counted (Table 3) and expressed as percentages (for absolute numbers, see Supplemental S6). There was a clear trend towards higher severity in the post-intervention procedure in the CCl_4_ group (also see the “intervention (post)” coefficient in model II). Here, the severity in the post-intervention was always higher than before an intervention (Χ^2^_CCl4_37.15, df = 4, *p* ≤ 0.001, with p_adj,mild/moderate_ =  ≤ 0.001, p_adj,mild/severe_ ≤ 0.001, p_adj,moderate/severe_ ≤ 0.006). In the control group (Oil) this was only found in the mild severity class (Χ^2^_Oil_ = 10.579, df = 4, *p* = 0.03, with p_adj,mild/moderate_ =  ≤ 0.044, p_adj,mild/severe_ ≤ 0.285, p_adj,moderate/severe_ ≤ 0.627).

**Table 3:** Severity class distribution in the treatment and intervention groups.

Treatment	Intervention	Counts per severity class			Total
		Mild	Moderate	Severe	
Oil	Bsl	11	7	0	18
Oil	Pre	93	41	2	136
Oil	Post	63	59	5	127
CCL4	Bsl	8	12	0	20
CCL4	Pre	49	54	3	106
CCL4	Post	9	69	13	91
Sum		233	242	23	498

**Figure 5:**
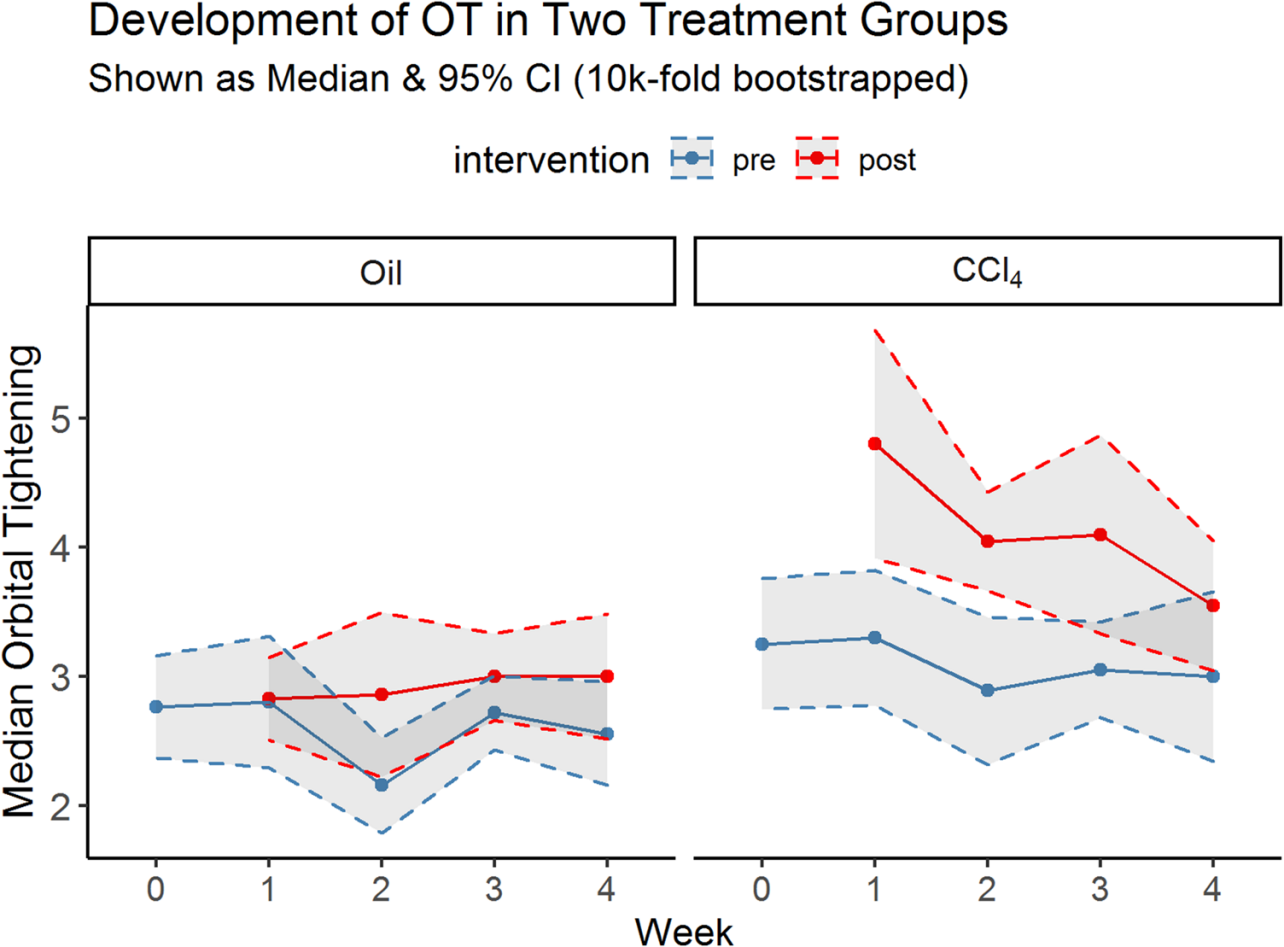
Bootstrapped (10 k-fold) estimates of the OT score in the two treatment regimens are shown as medians with 95% CI bands. The estimates in the control group showed no significant differences over time (overlapping confidence bands). There was also no difference regarding the intervention. In the CCl_4_ group, there were multiple differences in Orbital Tightening concerning the baseline values (week 0), time, and interventions. Intervention peaks in week 1, followed by a continuous return towards the untreated levels (negative slope) in week 4 as indicated by model II.

Orbital Tightening data were summarized and grouped by “treatment” and “intervention”. Since the orbital tightening variable showed mixed distributions over time (Supplemental Material S7) and the time-independent distribution was also not normally distributed (Shapiro Wilk’s test, *p* < 0.0001), value development was characterized as medians using a 10,000-fold bootstrapping from which also the 95% confidence intervals were obtained. The treatment-based medians were depicted and grouped by the intervention (“pre” (steel blue) / “post” (red)), and the corresponding confidence bands (Fig. 5). Week 0 had no injected animals and served as baseline measurement in both treatments. The control group showed no significant difference between the animals at the baseline and after the intervention (week 0 vs 1, W = 291.5, *p* value = 0.7875, *r*_control_ = 0.04). However, in the CCl_4_ group, a significant difference after treatment between weeks 0 and 1 was found, resulting in a medium-sized effect (W = 62.5, *p* value < 0.0001, *r*_CCL4_ = 0.64) and was considered highly significant.

## Discussion

This study aims at the possibility of simplification of the MGS to assess severity and pain level detection in mice. Our research aimed to evaluate the different MGS criteria and the potential simplification of its application, mainly to achieve a faster and more widespread implementation. Various criticisms were raised in assessing the MGS method concerning the effects on standard deviation and variance of the different facial criteria^6,21^. Overall, the OT-MGS model shows a high residual variance. However, an increased variance is often reported in behavioral studies^35^. For example, Hohlbaum et al. stated that an increased interrater variability causes the results to fluctuate, resulting in a high standard deviation^21^. In their recently published study, it was shown that the interrater variability is primarily also dependent on the examination criterion. There it was reported that the best agreement took place with the orbital tightening criterion, while the lowest agreements were achieved with nose and cheek bulge. In earlier studies, we were also able to identify gradations in the recognisability of the different criteria^15^. In general, these earlier studies had shown that there were no significant differences between or within raters when they were experienced. Despite this, the different criteria cannot be recognized with equal ease. The research of Cohen and Beths^20^ gives a good overview in their review of the use of the Grimace Scales in different animal species. Looking at their reappraisals, it becomes clear that mainly criteria for changes in the orbital tightening, ear, and nose are selected for assessment across all animal species. Taking together the results from the literature as well as the results of our study, the conclusion can be drawn that the orbital tightening criterion is a critical parameter in the MGS.

On the one hand, orbital tightening indicates to be the best discernible parameter^21^, and on the other hand, it has the strongest influence on the MGS score (Fig. 1, Table 1) in our study. This finding was demonstrated in two independent analyses, using the MoBPs algorithm (Fig. 1) and the penalized least square regression (Fig. 2). Both approaches confirmed each treatment group's parameter rankings (and their combinations).

Although automation by image processing and scoring algorithms is strongly demanded^3^ and pushed forward^36–38^, equal inclusion of all criteria is not yet feasible. Considering the various challenges in parameter recognition, the lack of feasibility in automation, and the high effort required to examine all criteria, the question of simplification arises. Consequently, and if automation is sought, there will be a need to use simplified evaluation criteria. From our experience, which is also confirmed by the investigation of the study by Hohlbaum et al., nose bulge and whisker change, for example, are criteria that are often not reliably assessed by both, experienced raters and algorithms having a slightly to moderate ease to judge, depending on the experimental setting. Our approach examined exemplarily the impact of the individual scoring criteria for the total score or the assignment of an animal to a discrete severity level. On this basis, and the observation that rating orbital tightening is the most reproducible^21^ as well as the most reliably identified criterion for evaluation (Figs. 1, 2), it was selected as the assessment parameter for further investigations in our study.

In the results presented in Fig. 1, we show that the orbital tightening parameter has the highest impact on the overall score, while whisker change has the least impact. While Table 1 indicatea high correlation between the individual parameters, it was confirmed in both groups (Fig. 2A, B) that orbital tightening ranked highest. The orbital tightening criterion mainly indicates differences in the intervention (Fig. 3A, B), especially in the CCl_4_ group after treatment (Fig. 3A), which as an expected pain stimulus and, therefore, was of particular interest in the investigation. Thus, we conclude that orbital tightening is a meaningful criterion in the grimace scale for investigating acute pain stimuli in our animal model. Rating of orbital tightening can discriminate differences between two treatment groups over time (Fig. 4A). As a pain stimulus, the injection itself and also the influence of the treatment (CCl_4_ vs oil) were studied over four weeks. However, significant differences in the baseline values of the treatment groups can be observed. Hence, the significance of the results between the treatment groups is diminished, indicating the limitations of this study.

By examining the distribution of the assessment data in the severity classes (Fig. 4), we can show that baseline values mostly result in a maximum to mild and occasionally a medium degree of orbital tightening. With the start of the treatments in week 1, an apparent increase in severity was given. Hence, the recognition of a clear acute pain stimulus in this model was seen (Fig. 4A). While single animals in the oil group also showed severe facial expressions in orbital tightening, this was seen in the CCl_4_ group in up to 14% of the cases after an intervention (Fig. 4B). This shows that the cumulation of pain compared to baseline is caused by both, the intervention of the *i.p.* injection (oil group) and by the injected substance itself, independently of time.

The development of the bootstrapped median severity estimates pre-and post-treatment of the two groups over time with their 95% confidence interval is shown in Fig. 5. The estimates in the control group showed no significant differences over time. We were able to show that the injection of CCl_4_ has an impact on the degree of pain and can be considered, in general, a model with moderate severity (Fig. 5). Even though the cumulative severity in the severe CCl_4_ class (Fig. 4B) was elevated from 2.3% to 13.8%, the largest shift took place in the moderate class. Here, a shift of 30% was observed (51.6% to 76.6%). There was no indication that the treatments or interventions caused severe pain. Instead, there was a moderate shift away from the mild class towards the moderate class. Nevertheless, some animals also showed a short-term severe orbital tightening behavior, which cannot, however, be explained with the treatment or time variable.

An overlap in confidence intervals in Fig. 5 indicates that the respective comparison showed no evidence for differences. If we look at the CCl_4_ group in detail, we see increased values shortly after the injection, especially in weeks 2 and 3. This indicates a painful impulse caused by the injection, which lasts over the investigation period of one hour after injection. These findings are in line with our recently published study on the severity of the CCl_4_ model itself^23^, which showed the highest severity of the animals in various clinical and behavioral parameters also during the second week of treatment. In Fig. 5 it was also demonstrated that the animals in the control group receiving only oil injections showed only a mild to moderate degree of severity in the orbital tightening scores. We can show that there is a high positive slope within the CCl_4_ group, which is most evident at the first and second weeks of treatment (Fig. 5). However, in the intervention of the control group, the pain stimulus did not seem to be caused by the medication but only by the intervention itself. The pain stimulus triggered by the injection alone did not seem to lead to either cumulative or habituation effects at these intervals. However, the negative slope in the post-intervention CCl_4_ group (Fig. 5) leads to smaller differences between pre-and post-intervention states over time. Consequently, the continuous decrease in the *within-subjects* intervention differences points towards a certain habituation effect in the CCl_4_ group. Although not significant, a decreasing effect of intervention severity over time (Fig. 3B) is perceivable, also supporting evidence for a possible habituation effect. However, this habituation effect in the CCl_4_ group may be due to the increased liver metabolism in the turnover of toxic CCl_4_ with the second week of treatment. These changes in liver metabolism were shown elsewhere by blood analysis in the CCl_4_ model^23^.

## Conclusion

Our study shows that in the present experimental setting, the examination with the primary focus on orbital tightening yields satisfactory results for the assessment of the degree of severity and for the inter-treatment group analyses. Considering these results, it can be concluded that this simplification of the MGS is feasible for practical use. We suggest that this can lead to faster applicability, a more straightforward automated procedure, and more quickly obtainable results. This is made possible because of better recognizability of the orbital tightening parameter, increasing reproducibility due to an increase in precision. Furthermore, a quick and simplified application is necessary when the MGS procedure is applied to more immediate settings, which can also serve as a potential target for refinement measures. To futher secure the statement of generalizability, the presented concept herein will have to be applied, tested, and verified with other studies, thereby gathering evidence that the pain stimulus shown in orbital tightening can also be detected in other stimuli and is not animal-model dependent.

The simplification procedure provides a basis for quick decision-making support and a further improvement in the quality of care. It may also offer options to facilitate automated monitoring procedures. At the same time, the MGS scoring in this study demonstrated that the severity caused by intraperitoneal injections was mainly dependent on the injected substance and not necessarily on the number of injections or the injection interval.

## Supplementary Information

Supplementary Information 1.

Supplementary Information 2.
