# Supplementary material for: A model-specific simplification of the Mouse Grimace Scale based on the pain response of intraperitoneal CCl4 injections
Source: Sci Rep. 2022 Jun 28;12:10910. doi: 10.1038/s41598-022-14852-0 (PMC9240072; doi:10.1038/s41598-022-14852-0)
Supplement: Supplementary file 2 — Supplementary Information 2. [file 41598_2022_14852_MOESM2_ESM.pdf]

Supplemental Material

S1 Model descriptions, usage and interpretation

Generalized linear models (or regressions) can be used in the characterization of treatment contrasts. In such models, the default factor levels are part of the intercept and the coefficients indicate factor level development as linear contrasts with regard to the dependent variable. We implemented this statistical method to quantify the factor dependencies on the orbital tightening variable (OT) as regression coefficients. The estimates of each regression are shown in separate tables below.

The influence of each factor is coded in the model coefficients (β). With these estimates, the linear effect of changing one unit in the current factor on the dependent variable is shown. For example, in Model II - the *within-treatment* model for CCl_4 - the coefficient for the intervention[post] variable shows:

$$\beta_{\text{intervention}} = 1.73,\ CI_{95\%}[1.12;\ 2.34],\ p<0.001$$

This can be interpreted as follows: Disregarding all other factors in the model, the general effect of an intervention (post) against the default level of "pre" in the intercept ($\beta_{intercept}$=3.3) shows a 1.73 units increase in orbital tightening. This effect is significant. Therefore, the intervention has an overall effect in the CCl_4 group. In contrast, the Oil-group shows the same analysis as (intervention[post]):

$$\beta_{\text{intervention}} = 0.25,\ CI_{95\%}[-0.22;\ 0.71],\ p=0.293$$

Here, the estimate is not only much smaller but it is also not significant. This means that there is no general intervention effect within the Oil-treatment group.

Depending on the approach, each of the three models shows different coefficients and, therefore, has a different meaning. A *between-treatments* model (e.g., Oil vs CCl_4 treatment shows other coefficients than a *within-treatment* model (e.g, just CCL_4)) – especially, when the rank deficient week 0 is excluded from the analysis. Then the default level of week is no longer 0 but 1.

The complexity of such treatment contrasts allows for different analyses and generalizations. On the one hand, we were interested in a general quantification of differences between treatments and, of course, the effect of continuous interventions over time within each treatment. Since two time resolutions were available, they were modeled independently. In the highest available resolution (day), they were also modeled as nested random effects to explain more of the unexplained variance.

To explain the data sufficiently, we addressed this need by using three different linear mixed-effects regressions. The models were adapted and solved as efficiently as possible. This was also the reason, why Models II and III did not contain nested time effects, or a day:week-interaction: the scaled gradient of the Hessian was not obtainable with this structure and the model was not able to converge. Thus, the models were simplified to the next higher time-frame, e.g., weeks.

S2 Model overview

Model I A general *between-treatments* model with a random intercepts term for animal ID as well as the time variable day nested in week.

OT ~ treatment : day : intervention + (1|ID) + (1|week/day)

The model analyses time-dependent differences between treatments and uses the nested random effect (1|week/day) to explain more of the remaining variance in the data. The contrasts were, therefore, always reported to the default levels in the treatment group (Oil) intercept. The default contrasts were: treatment=Oil, day = 1, week=0, intervention=pre.

Model II A general *within-treatment* model for CCl_4 with a random intercept term for the animal ID.

OT ~ week : intervention + (1|animal_id)

In this model, week=0 data were excluded, because there were no data for the intervention variable in that week. To avoid rank-deficiency errors, the baseline comparisons were calculated separately. Due to the exclusion of week=0 data, the default contrasts changed: week=1, intervention=pre.

Model III The same as in Model II but with the Oil group (control).

Linear Models - Output

The following table list the coefficient output of the model summaries. Significant coefficients are highlighted.

S3 Model I ***Between-treatment*** **analysis of Oil vs. CCl_4.**

OT ~ treatment : day : intervention + (1|ID) + (1|week/day)

	OT			
Predictors	*Estimates*	*CI*	*p*	*df*
(Intercept)	2.59	2.04 – 3.14	**<0.001**	34.88
treatment [CCL4]	0.61	-0.05 – 1.26	0.068	57.98
day [2]	-0.18	-0.80 – 0.45	0.554	14.75
day [3]	0.34	-0.29 – 0.98	0.267	15.98
intervention [post]	0.52	0.07 – 0.96	**0.022**	419.79
treatment [CCL4] * day [2]	0.43	-0.21 – 1.06	0.187	418.71
treatment [CCL4] * day [3]	-0.25	-0.91 – 0.42	0.467	419.23
treatment [CCL4] * intervention [post]	1.03	0.36 – 1.70	**0.003**	419.49
day [2] * intervention [post]	0.02	-0.59 – 0.63	0.948	418.55
day [3] * intervention [post]	-0.49	-1.13 – 0.15	0.133	419.20
(treatment [CCL4] * day [2]) * intervention [post]	-0.43	-1.36 – 0.51	0.372	419.05
(treatment [CCL4] * day [3]) * intervention [post]	0.19	-0.78 – 1.16	0.702	419.85
**Random Effects**				
σ^2	1.09			
$\tau_{00\ animal\_id}$	0.32			
$\tau_{00\ day:week}$	0.08			
$\tau_{00\ week}$	0.01			
ICC	0.28			
N_{animal_id}	24			
N_{day}	3			
N_{week}	4			
Observations	460			
Marginal R^2 / Conditional R^2	0.258 / 0.464			

S4 Model II ***Within-treatment*** **analysis of CCl_4.**

OT ~ week : intervention + (1|animal_id)

	OT			
Predictors	*Estimates*	*CI*	*p*	*df*
(Intercept)	3.30	2.77 – 3.83	**<0.001**	36.55
week [2]	0.00	-0.60 – 0.60	0.991	179.59
week [3]	0.13	-0.49 – 0.76	0.676	182.05
week [4]	-0.18	-0.84 – 0.49	0.600	182.65
intervention [post]	1.73	1.12 – 2.34	**<0.001**	178.95
week [2] * intervention [post]	-0.57	-1.47 – 0.33	0.210	178.65
week [3] * intervention [post]	-0.40	-1.31 – 0.51	0.391	178.44
week [4] * intervention [post]	-0.75	-1.73 – 0.22	0.128	178.50
**Random Effects**				
σ^2	1.39			
$\tau_{00\ animal\_id}$	0.34			
ICC	0.20			
N_{animal_id}	12			
Observations	197			
Marginal R^2 / Conditional R^2	0.223 / 0.376			

S5 Model III ***Within-treatment*** **analysis of Oil.**

OT ~ week : intervention + (1|animal_id)

	OT			
Predictors	*Estimates*	*CI*	*p*	*df*
(Intercept)	2.70	2.24 – 3.15	**<0.001**	36.96
week [2]	-0.34	-0.80 – 0.13	0.152	244.16
week [3]	0.33	-0.12 – 0.79	0.151	244.05
week [4]	-0.28	-0.76 – 0.19	0.238	244.14
intervention [post]	0.25	-0.22 – 0.71	0.293	244.16
week [2] * intervention [post]	0.38	-0.31 – 1.07	0.282	244.49
week [3] * intervention [post]	0.01	-0.64 – 0.66	0.978	244.08
week [4] * intervention [post]	0.11	-0.56 – 0.77	0.754	244.11
**Random Effects**				
σ^2	0.95			
$\tau_{00\ animal\_id}$	0.28			
ICC	0.23			
N_{animal_id}	12			
Observations	263			
Marginal R^2 / Conditional R^2	0.068 / 0.282			

Table S6. Absolute numbers of data points in the severity classes.

		Severity class			
**treatment**	**intervention**	**mild**	**moderate**	**severe**	**total**
**Oil**	**bsl**	11	7	0	**18**
**Oil**	**pre**	93	41	2	**136**
**Oil**	**post**	63	59	5	**127**
**CCL4**	**bsl**	8	12	0	**20**
**CCL4**	**pre**	49	54	3	**106**
**CCL4**	**post**	9	69	13	**91**
**Sum**		**233**	**242**	**23**	**498**

S7 Distributions

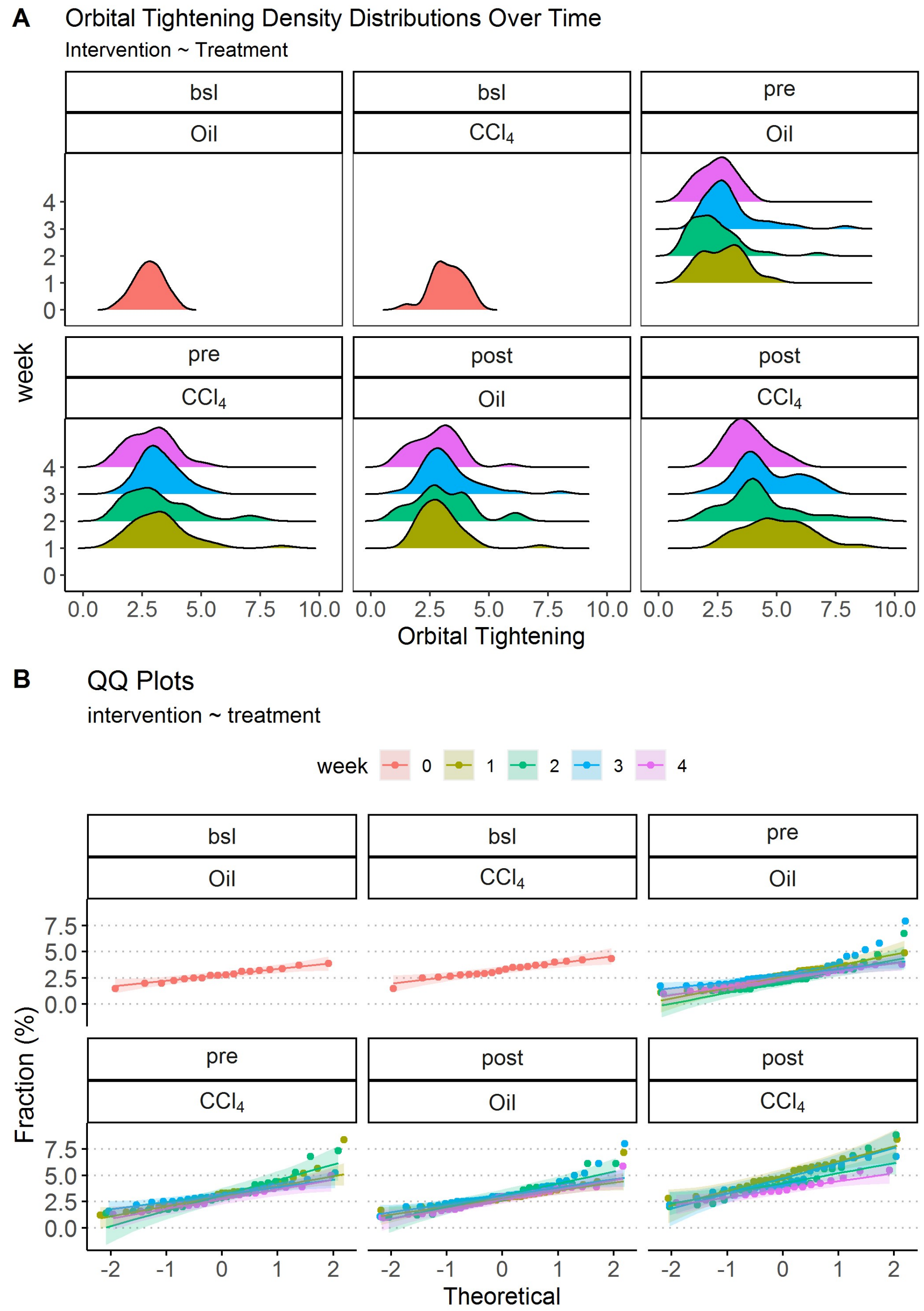

Figure S1. *(**A**) The density distributions show variable distributions over time. At baseline (bsl) on week 0 in the Oil treatment group, data show a normal distribution. In contrast, other data suggest severe deviations from normality, e.g., with strong tailings towards higher orbital tightening values or bimodal behavior (e.g., see pre/CCl_4 in week 2). Due to the mixed nature of the data, a non-parametric bootstrapping was chosen to find conditional estimates. (**B**) The group-dependent deviations from normally distributed data were confirmed by visual inspection of the respective QQPlots. In both figures, 489 cases with n=24 animals contributed to the plots.*
